# Evidence Based Practice Attributes Across Nursing Roles in A Children's Hospital

**DOI:** 10.1111/wvn.70020

**Published:** 2025-04-03

**Authors:** Rose Chapman‐Rodriguez, Reynaldo Rivera, Joyce Fitzpatrick

**Affiliations:** ^1^ Morgan Stanley Children's Hospital‐New York Presbyterian Hospital New York New York USA; ^2^ Frances Bolton School of Nursing Case Western University Cleveland Ohio USA

**Keywords:** advanced practice registered nurses, beliefs, evidence‐based practice, implementation science, nurse leaders, nursing roles, organizational culture, pediatrics, unit culture

## Abstract

**Background:**

Evidence‐based practice (EBP) attributes are associated with improved patient care outcomes. There is a paucity of knowledge on pediatric nurses' attributes based on their clinical sub‐specialties.

**Aim:**

To investigate the relationships between pediatric nurses' EBP attributes and background variables, including their academic degree, years of experience, and clinical specialty.

**Methods:**

A convenience sample of 185 nurses participated in this descriptive, cross‐sectional study. The electronic surveys included 11 background questions and the short‐versions of the EBP Beliefs Scale, Organizational Culture and Readiness Scale, and EBP Implementation Scale.

**Results:**

EBP belief scores were notably higher in pediatric nurses in neonatology, critical care, and among nurse leaders. No statistically significant difference was found in EBP organizational culture among nurse leaders, clinical nurses, and advanced practice nurses. EBP implementation was favorable in neonatology, acute care, and nurse leaders. No significant results were found in EBP attribute scores related to nurses' age, academic nursing degree, or years of experience.

**Linking Evidence to Action:**

This study confirmed findings from prior studies acknowledging the impact nurse leaders have on creating and sustaining a favorable EBP culture and implementation science. Organizational attributes such as Magnet status, a shared governance structure, support for specialty certification, and EBP mentorship also reinforce nursing EBP attributes. Further research should investigate unit‐level strategies and measure the impact on pediatric patient care outcomes.

## 1 Introduction

Evidence Based Practice (EBP), a problem‐solving approach to the delivery of health care, has received considerable attention in nursing research and professional practice. Further, EBP attributes including nurses' beliefs, knowledge, and skills have been shown to be positively associated with increased EBP implementation and improved patient outcomes (Carrol et al. 2021; Yoo et al. 2019).

In prior studies, shared professional governance, local unit culture, EBP mentors, and professional specialty certification were identified as factors contributing to greater EBP implementation (Alves 2021; Djukic et al. 2021; Jun et al. 2020). Nurse leaders and managers influence clinical nurses' EBP beliefs, culture, and implementation (Caramanica et al. 2022; Nelson‐Brantley and Chipps 2021; Shuman et al. 2020). Advanced practice nurses (APNs) and doctoral‐prepared nurses were also identified as expert EBP coaches for nurses (Clarke et al. 2021; Dagne and Beshah 2021; McNett et al. 2021). Recent studies in adult patient care units show nurses have high EBP awareness and knowledge but exhibit poor EBP implementation science (Furtado et al. 2024; Zhang et al. 2024).

Most of the nursing research on EBP attributes was conducted with nurses and students working in adult care settings (Cleary‐Holdforth et al. 2021; Engels et al. 2020; Labrague et al. 2019). Little is known of the EBP attributes of pediatric clinical nurses, advanced practice nurses, and nurse managers (Bell 2020). The only previous EBP study with pediatric nurses was completed in 2018. In this study of 190 pediatric nurses across the United States, Connor (2018) found nurses had moderate EBP knowledge and minimal experience implementing EBP.

### 1.1 Aim of Study

The purpose of the study was to describe EBP beliefs, organizational culture, and implementation across all levels of pediatric nurses in a free‐standing children's hospital. Differences based on highest academic nursing degree, years of experience, and clinical sub‐specialty were also examined.

## 2 Background

A comprehensive literature search was performed in CINAHL, PubMed, and EBSCO databases from the years 2019 through 2024. Out of 1100 research articles, 12 were reviewed and selected based on their alignment with this studies' focus. The concepts reviewed within this search were EBP, attributes, pediatrics, beliefs, organizational culture, EBP implementation science, and nursing roles.

### 2.1 EBP Beliefs

Clinicians self‐report low EBP beliefs, insufficient knowledge, educational training programs, minimal or no support from their leaders, and not having a full understanding of EBP culture and implementation science at the organizational level (Lam et al. 2020). Barriers to productive EBP organizational environments in the USA include nurses that do not believe EBP is important or that EBP adds value to patient care outcomes (Crable et al. 2021). In addition, inadequate access to library resources, mentors, and organizational challenges, such as the inability to provide protected time, also hinder EBP culture and effective attributes (Duff et al. 2020).

Some variables associated with EBP success are professional role, clinical specialty, professional nursing certification, competency, implementation of EBP in the past, access to EBP mentors, collaborative team approach, educational training programs, community engagement, shared decision model, Magnet status, and institutional policy (Connor 2018; Gallagher‐Ford et al. 2020).

EBP beliefs were measured with the Evidence‐based Practice Beliefs Scale (shortened version) which is a 3‐item Likert scale from 1 (*strongly disagree*) which carries a minimum score of 1.0 for all questions in the scale to 5 (*strongly agree*) with a maximum score of 5. Sample questions include “I believe that EBP results in the best clinical care for patients,” “I am sure that I can implement EBP,” and “I am sure that implementing EBP will improve the care that I deliver to my patients” (Melnyk et al. 2021a; Osuchukwu and Ezeruigbo 2023).

### 2.2 EBP Culture

Duff et al. (2020) conducted semi‐structured, in‐depth interviews with 12 participants (nurse scientists, faculty, and managers) experienced with the IOWA EBP model. This study showed the value of a shared EBP model using an interdisciplinary collaborative approach, with implementation guidance, intense EBP training, nurse manager engagement, and the presence of nurse scientists. Huo et al. (2024) found EBP competence, intention to implement, and work control, influenced the organizational climate on evidence‐based practice behavior with their descriptive survey. The Evidence‐based Practice Culture and Environment Scale (short version) measures the extent to which cultural factors influence system‐wide implementation of EBP and overall perceived readiness for EBP integration. The 5‐point Likert scale includes the following: “My organization has a culture that supports clinicians to implement EBP,” “My organization has readily available resources to implement EBP,” My organization provides EBP mentors to assist clinicians in implementing EBP (Melnyk et al. 2021a; Osuchukwu and Ezeruigbo 2023).

### 2.3 EBP Implementation

Yoo et al. (2019) conducted a descriptive, cross‐sectional study with a sample of 485 nurses working with adult patients in South Korea. EBP implementation was significantly correlated with EBP knowledge and organizational readiness. Melnyk et al. (2021a) conducted a structural equation model to test the relationship with several variables and found EBP culture and mentorship had a positive impact on EBP knowledge, beliefs, competency, implementation, job satisfaction, and intent to stay.

Jun et al. (2020) researched the relationship between nursing unit culture and EBP implementation. Group culture was the most common (47.3%) culture associated with the practice of EBP. Interprofessional units function effectively and are more likely to support strong EBP environments when they display social behaviors such as: collaborate, commit, communicate, facilitate, mentor, build teams, participate, and drive human development (Jun et al. 2020).

Having a master's degree or higher and prior experience conducting research had a favorable impact on the level of EBP implementation (Furuki et al. 2022). Evidence‐based Practice Implementation Inventory (short version) measures the application of EBP into practice. Sample questions include, “I use evidence to improve patient outcomes in my healthcare setting,” “I implement the steps of the EBP process in my practice,” and “I promote the use of EBP in my healthcare setting to improve outcomes” (Melnyk et al. 2021a).

### 2.4 Nursing Roles

Clarke et al. (2021) evaluated nurse practitioner (NP) beliefs, level of EBP implementation, and barriers in a scoping review from 2009 to 2018. They found NPs value EBP, but implementation was low as they reported a lack of time, competence, and support from colleagues and their managers. One of the most influential nursing roles affecting clinical nurses' EBP beliefs, culture, and implementation is the nurse manager and leader (Nelson‐Brantley and Chipps 2021; Shuman et al. 2020).

## 3 Methods

### 3.1 Research Design

A descriptive, cross‐sectional study was conducted with electronic surveys. All nurses employed in a free‐standing pediatric hospital were eligible to participate (*N* = 1243). Institutional Review Board approval was obtained through an expedited review prior to enrolling participants.

### 3.2 Determination of Sample Size/Power Analysis

Using the G Power statistical program to estimate sample size for up to five groups with the categories nursing roles and academic degree (Table 1). One‐Way ANOVA analysis was based on parameters: *p*‐value of 0.05, power of 0.8, assuming a medium effect size of 0.25, and a minimum number of 159 participants was needed to detect statistical significance (Erdfelder et al. 2009).

**TABLE 1:** Evidence‐based practice research variables defined (dependent and independent variables).

Demographics/independent variables		
Academic nursing degree	Categorical	Bachelor of Arts, Master of Science, Doctor of Nursing Practice (DNP)/Doctor of Nursing Science (PhD)
Pediatric subspecialty	Categorical	Acute care, ambulatory care, critical care, obstetrics, neonatology, leader, other
National Board Certification	Dichotomous	Yes or no
Nursing Role	Categorical	Clinical Nurse, Advanced Practice Nurse (nurse educator, nurse practitioner, clinical nurse specialist, nurse midwife), Nurse Manager (clinical nurse manager, patient care director, director of nursing, chief nurse office, nurse scientist)
Years of experience	Continuous	Number of years

Dependent variables		
EBP belief scale	Interval data	3 separate Likert questions (1–5)
EBP implementation scale	Interval data	3 separate Likert questions (1–5)
EBP organizational culture scale	Interval data	3 separate Likert questions (1–5)

### 3.3 Procedures

All per diem, part‐time, and full‐time nurses currently employed at the hospital in all nursing roles were recruited. A global, nursing‐wide email listserv along with flyers with a QR code to the survey link were posted on every unit. From April 10th, 2023, through May 15th, 2023, four weekly email reminders were sent by the Enterprise Director of EBP, Research, and Innovation to avoid recruitment coercion from the lead investigator. Qualtrics data was anonymously collected, participant data were checked for missing demographic or survey responses, and data were cleaned before entering in a secure data file. Qualtrics data was converted into SPSS file for statistical analysis in SPSS version 25 software. An outline of the dependent and independent variables is provided (Table 1).

### 3.4 Instruments

A survey tool was created with 11 questions asking participant age, ethnicity, nursing role, years of experience, national nursing board certification, academic degree, specialty area, and prior exposure to EBP. The short version of the EBP Beliefs Scale, EBP Implementation Scale, and Organizational Culture and Readiness Scale for System‐wide Integration of Evidence‐Based Practice (OCRSIP) Scale developed by Melnyk et al. (2021b) was used to measure the EBP dependent variables (beliefs, culture, and implementation).

The three shortened scales had convergent validity: EBP Beliefs (*r* = 0.64, *p* < 0.001), EBP Implementation (*r* = 0.42, *p* < 0.001), and OCRSIEP Scale (*r* = 0.72, *p* < 0.001). Cronbach's *α* for the shorted EBP Beliefs scale is 0.81, shorted EBP Implementation scale = 0.89, and shorted OCRSIEP scale = 0.87. The Cronbach's α for this current study were EBP beliefs scale (0.86), EBP Culture (0.88), and EBP Implementation (0.91).

## 4 Results

Of the 1243 nurses eligible for the study, 217 participants responded to the survey; of these, 185 nurses were included in the final sample for a response rate of 15%. Most participants were female nurses (91.9%) with White ethnicity (51.4%) and a mean age of 40.41 years (SD = 12.54). Clinical nurses made up 69.2% of respondents, APNs comprised 18.9%, and nurse leaders were 11.9%. One hundred and twenty‐seven participants indicated they were nationally certified in a nursing specialty (68.6%), bachelor's degree (54.6%), master's degree (38.4%), or doctoral degree (6.5%) (Table 2).

**TABLE 2:** Descriptive characteristics of participants.

	Frequency	Percent
*Gender*		
Male	12	6.5
Female	170	91.9
Non‐binary/third gender	2	1.1
Prefer not to say	1	0.5
*Race/ethnicity*		
White	95	51.4
Black or African American	10	5.4
Asian	45	24.3
Hispanic or Latino	35	18.9
*Current nursing role*		
Clinical nurse	128	69.2
Advanced practice nurse	35	18.9
Nurse leader	22	11.9
*Employment status*		
Full‐time	173	93.5
Part‐time	10	5.4
Per diem	2	1.1
*Are you nationally certified in a nursing specialty?*		
No	58	31.4
Yes	127	68.6
*Degree*		
Doctorate (DNP or PhD)	12	6.5
Masters	71	38.4
Bachelors	101	54.6
Associates	1	0.5
*Nursing specialty at MSCH*		
Neonatology	53	28.6
Pediatric acute care	34	18.4
Pediatric critical care	28	15.1
Pediatric ambulatory care	20	10.8
Obstetrics	28	15.1
Nurse leader/director	10	5.4
Other	12	6.5
*What has been your exposure to the concept of evidence‐based practice*? (*Check all that apply*)		
Learned about EBP in nursing school	149	42.82
Member of a project team in the past or presently engaged in an EBP project	79	22.7
Member of professional shared governance council involved in EBP	53	15.23
Took a workshop or training program in EBP	48	13.79
Presence of an EBP Mentor	12	3.45
Do not know much about EBP	7	2.01

Abbreviations: DNP, doctor of nursing practice; EBP, evidence‐based practice; MSCH, Morgan Stanley Children's Hospital.

Among participants, 42.82% reported learning about EBP in nursing school, 12 individuals (3.45%) mentioned having an EBP mentor, and 7 nurses reported not having much knowledge about EBP. The youngest age was 22 years old and the oldest age was 69 years, showing a broad generational gap (Table 2). The respondents had approximately 19.44 years of experience (SD = 18.11), showing a significant dispersion of years of experience (Table 3).

**TABLE 3:** Sample characteristics: Continuous variables.

	Mean	Standard deviation
Age in years	40.4108	12.54
Years of experience	19.44	18.12
EBP belief mean scores	4.24	0.99
EBP organizational culture mean scores	3.42	1.05
EBP implementation mean scores	4.02	0.83

Abbreviation: EBP, evidence‐based practice.

### 4.1 Research Question 1: What Are the EBP Attributes in Pediatric Nurses Based on Their Subspecialty?

Using descriptive statistics, EBP belief mean scores of pediatric nurses across the sub‐specialty roles were as follows: pediatric critical care nurses (*M* = 4.47) and neonatal nurses displayed high mean scores (*M* = 4.33), indicating a strong belief in EBP. Pediatric acute care nurses (*M* = 4.10) and pediatric ambulatory care nurses (*M* = 4.21) had mean scores suggesting they agreed with having a positive attitude toward EBP in their practice. Obstetrics nurses (*m* = 3.97) indicated a lower but still favorable belief in EBP. Nurse leaders displayed the highest mean score of 4.50. Nurses in the “other” category included lactation consultants, wound ostomy nurses, nurse educators, and quality improvement analysts scored lowest (*M* = 3.25) (Table 4).

**TABLE 4:** Evidence‐based practice (EBP) attributes of participants.

		Mean	Standard deviation	Standard error
EBP Belief mean scores	Neonatology	4.33	0.97	0.13
	Pediatric acute care	4.10	0.94	0.16
	Pediatric critical care	4.47	0.46	0.09
	Pediatric ambulatory care	4.21	0.83	0.18
	Obstetrics	3.97	1.47	0.27
	Nurse leader/director	4.50	0.57	0.18
	Other	4.25	1.22	0.35
EBP organizational culture mean scores	Neonatology	3.17	1.12	0.15
	Pediatric acute care	3.63	0.73	0.13
	Pediatric critical care	3.38	0.96	0.18
	Pediatric ambulatory care	3.61	1.08	0.24
	Obstetrics	3.24	1.25	0.24
	Nurse leader/director	4.30	0.65	0.20
	Other	3.27	1.06	0.32
EBP implementation mean scores	Neonatology	4.20	0.67	0.09
	Pediatric acute care	4.05	0.77	0.14
	Pediatric critical care	3.88	0.67	0.13
	Pediatric ambulatory care	3.85	0.56	0.13
	Obstetrics	3.83	1.26	0.24
	Nurse leader/director	4.33	0.52	0.16
	Other	3.84	1.17	0.35

The EBP organizational culture of pediatric nurses was examined across different sub‐specialty roles. Neonatology nurses (*M* = 3.17) and pediatric critical care nurses (*M* = 3.38) showed a neutral‐moderate level of EBP culture in their sub‐specialty. Pediatric acute care nurses (*M* = 3.63) and pediatric ambulatory care nurses (*M* = 3.61) showed a stronger EBP culture within their care settings. Obstetrics nurses exhibited a mean score of 3.24, suggesting a moderate level of EBP organizational culture. Nurse leaders and managers (*m* = 4.30) demonstrated a strong EBP culture and support within their leadership roles.

The EBP implementation mean scores of pediatric nurses were examined. Neonatology nurses (*m* = 4.20), and pediatric acute care nurses (*m* = 4.05) suggest a favorable level of EBP implementation within their practice environment. Pediatric critical care (*m* = 3.88), pediatric ambulatory care (*m* = 3.85), and obstetric nurses (*m* = 3.83) show a moderate level of EBP implementation. Nurse leaders and managers demonstrated a high mean score of 4.33, emphasizing a solid commitment to EBP implementation within their leadership roles. Nurses in the “other” category (*m* = 3.84) indicate moderate EBP implementation (Table 4).

### 4.2 Research Question 2: Are There Differences in EBP Beliefs, Implementation, and Organizational Culture Mean Scores Related to Nursing Roles, Academic Degrees, or Years of Experience?

A one‐way ANOVA was used, homogeneity of variance can be assumed using the Levene's test which showed variances in all groups are similar (Supporting Information Tables S1–S3). For all the variables, *p* value of > 0.05 validated this assumption. Skewness for the years of experience, the “EBP organizational culture mean scores” variable, and the “EBP implementation mean scores” variable was from −2 to +2 (Supporting Information, Table S4). There was no significant difference in EBP beliefs, organizational culture, or EBP implementation scores based on nurses' academic degrees or years of experience (Supporting Information, Tables S5 and S6). No significant difference was found in EBP belief and EBP implementation scores related to nursing roles (*F* (2,179) = 1.700, *p* = 0.186); (*F* (2,169) = 2.57, *p* = 0.079). There was a statistically significant difference in EBP organizational culture mean scores related to nursing roles (*F* (2,174) = 3.304, *p* = 0.039) (Supporting Information, Table S7). Post hoc analysis using Games‐Howell test for unequal group sizes, revealed nurse leaders (*M* = 3.95) had higher mean scores compared to clinical nurses (*M* = 3.34) and APNs (*M* = 3.34), but no significant differences were found between nurse leaders and clinical nurses (*p* = 0.053); APNs and nurse leaders (*p* = 0.174); or clinical nurses and APNs (*p* = 0.845) in regards to organizational culture and resources (Supporting Information, Table S8).

### 4.3 Summary of Findings

Nursing sub‐specialty and the nurse leader role in a children's hospital were key variables in higher EBP belief and implementation scores. EBP belief mean scores were notably higher in nurses working in neonatology (*M* = 4.33), critical care (*M* = 4.47), and among nurse leaders (*M* = 4.50). EBP implementation (*M* = 4.02) was favorable in neonatology (*M* = 4.2), acute care (*M* = 4.05), and nurse leaders (*M* = 4.33). There was no statistically significant difference in mean scores with EBP organizational culture; nurse leaders (*M* = 3.95), clinical nurses (*M* = 3.34), and APNs (*M* = 3.34). No significant difference was found in EBP belief, EBP organizational culture, or EBP implementation mean scores, based on nurses' years of experience in our cohort (EBP beliefs (*r* = −0.06, *p* = 0.400), organizational readiness (*r* = 0.02, *p* = 0.770), and implementation (*r* = 0.01, *p* = 0.867) (Supporting Information Table S9).

Overall participant EBP belief (*M* = 4.24) was high among the surveyed population with moderate variability. EBP organizational culture (*M* = 3.42) indicated a moderate level of organizational culture, suggesting a higher degree of variability in the EBP organizational culture scores compared to the belief scores across roles, ages, and specialty. EBP implementation (*M* = 4.02) showed a prominent level and moderate variability in the EBP implementation scores (Table 3).

## 5 Discussion

This is the only study to date that used the 3 EBP short version scales to evaluate attributes across nursing roles in an ethnically diverse Children's Hospital.

Nurses represented 50% White and 50% non‐Whites compared to prior EBP studies with 75%–95% white participants (Connor 2018; Melnyk et al. 2021a). Participants' age ranged from 22 to 69 years, showing a broad generational gap and maturity (*M* = 40.4). Nursing experience was about 20 years, which may reflect strong retention, prolonged organizational EBP exposure, and extensive experiential value.

EBP organizational culture measures the system‐wide resources within a healthcare system. EBP culture may differ at the micro‐unit level, compared to the macro—enterprise level (Melnyk et al. 2021a). The variability in our EBP mean scores based on sub‐specialty areas may reveal those differences in group culture at the unit‐level and local nurse leadership influence.

Unlike prior studies linking a master's degree or higher with improved EBP implementation (McNett et al. 2021; Nelson‐Brantley and Chipps 2021), we found that an academic nursing degree had no association with EBP attributes and implementation. Gigli et al. (2020) also found that the educational level in adult critical care nurses had no relationship to EBP beliefs or practices. This finding is reassuring since nurses at all levels showed strong EBP attributes regardless of having a bachelors, masters, or doctoral degree. EBP implementation was strong in our pediatric cohort nurses compared to studies referenced in the literature.

Shared professional governance models align nurses, patients, environments, and leaders on the mission of quality patient care (Williams and Christopher 2023). Targeted interventions to promote and sustain EBP culture may include mentors, local nurse‐leaders, team collaboration, along with organizational support (Cassidy et al. 2021). Several of the respondents had national certification, EBP exposure in school, or participated in unit projects which demonstrated favorable EBP attributes. We have seasoned and mature nurses in our Children's Hospital which may factor in stability and investment in EBP beliefs, culture, and implementation.

Several years past the COVID‐19 pandemic, people, communities, and health care systems all endured significant loss, change, staffing crisis, and a surge in onboarding new graduates (Forbes 2022). Based on our results, we have an opportunity to develop unit‐level EBP mentors with nurses interested in sustaining EBP culture. Despite the local, organizational, and global changes, it is reassuring to find that a sample of our nurses' values and upholds EBP attributes and are invested in creating an EBP framework that supports EBP implementation across all levels of nursing.

### 5.1 Key Points

- In addition to organizational culture, unit‐level group culture may be a factor in EBP nursing attributes.
- Nurse leaders demonstrate strong EBP attributes and have influence on local and organizational EBP culture and implementation.
- Opportunity exists for EBP mentorship programs.

### 5.2 Limitations

There were a few limitations that may have affected the outcomes of this research. We had a 15% response rate despite adequate power for analysis. The descriptive and analytical findings may be specific to the cohort studied in this Children's Hospital. As a cross‐sectional design, this study describes the population at one time point, and no inferences can be made.

#### 5.2.1 Linking Evidence to Action

- Unit—level group culture may be a factor in EBP nursing attributes.
- Nurse leaders show strong EBP attributes and have influence on local and organizational culture and implementation.
- The role of unit‐specific teams and their collaboration with leaders should be further investigated to understand the application of implementation science, patient care outcomes, and return on investment.

## 6 Conclusions

This study helps the organization identify nurse's EBP attributes across roles and subspecialties, which helps us in targeted interventions that support local unit culture where needed, and sustain the strong EBP beliefs, culture, and implementation shown in our findings. There are opportunities for greater mentoring support and continued organizational readiness and awareness at the local unit level, which may continue to enhance EBP implementation science across all nursing roles in our pediatric organization. Magnet status, shared governance, specialty certification, and nurse leaders play a significant role in favorable EBP culture and implementation.

## Conflicts of Interest

The authors declare no conflicts of interest.

## Supporting information




Data S1.

## Data Availability

The data that support the findings of this study are available from the corresponding author upon reasonable request.
