# Supplementary material for: Evidence Based Practice Attributes Across Nursing Roles in A Children's Hospital
Source: Worldviews Evid Based Nurs. 2025 Apr 3;22(2):e70020. doi: 10.1111/wvn.70020 (PMC11967156; doi:10.1111/wvn.70020)
Supplement: Supplementary file 1 — Data S1. [file WVN-22-0-s001.docx]

EVIDENCE BASED PRACTICE ATTRIBUTES ACROSS NURSING ROLES IN A CHILDRENS HOSPITAL

SUPPLEMENTAL MATERIAL

Tables with statistical analysis

**TABLE S1** Tests of homogeneity of variances - nursing roles

|  | | Levene Statistic | df1 | df2 | Sig. |
| --- | --- | --- | --- | --- | --- |
| EBP Belief Mean Scores | Based on Mean | .152 | 2 | 179 | .859 |
|  | Based on Median | .069 | 2 | 179 | .933 |
|  | Based on Median and with adjusted df | .069 | 2 | 166.018 | .933 |
|  | Based on trimmed mean | .122 | 2 | 179 | .885 |
| EBP Organizational Culture Mean Scores | Based on Mean | .548 | 2 | 174 | .579 |
|  | Based on Median | .292 | 2 | 174 | .747 |
|  | Based on Median and with adjusted df | .292 | 2 | 163.239 | .747 |
|  | Based on trimmed mean | .490 | 2 | 174 | .613 |
| EBP Implementation Mean Scores | Based on Mean | 1.151 | 2 | 169 | .319 |
|  | Based on Median | 1.070 | 2 | 169 | .345 |
|  | Based on Median and with adjusted df | 1.070 | 2 | 152.365 | .346 |
|  | Based on trimmed mean | .988 | 2 | 169 | .374 |

*Note*. EBP = evidence-based practice; Independent variable = nursing roles.

**TABLE S2** Tests of homogeneity of variances - nurses' academic degree.

|  | | Levene Statistic | df1 | df2 | Sig. |
| --- | --- | --- | --- | --- | --- |
| EBP Belief Mean Scores | Based on Mean | 1.685 | 2 | 178 | .188 |
|  | Based on Median | 1.384 | 2 | 178 | .253 |
|  | Based on Median and with adjusted df | 1.384 | 2 | 174.324 | .253 |
|  | Based on trimmed mean | 1.386 | 2 | 178 | .253 |
| EBP Organizational Culture Mean Scores | Based on Mean | .038 | 2 | 173 | .963 |
|  | Based on Median | .049 | 2 | 173 | .952 |
|  | Based on Median and with adjusted df | .049 | 2 | 172.759 | .952 |
|  | Based on trimmed mean | .022 | 2 | 173 | .978 |
| EBP Implementation Mean Scores | Based on Mean | 1.348 | 2 | 168 | .263 |
|  | Based on Median | 1.310 | 2 | 168 | .273 |
|  | Based on Median and with adjusted df | 1.310 | 2 | 151.399 | .273 |
|  | Based on trimmed mean | 1.113 | 2 | 168 | .331 |

*Note*. EBP = evidence-based practice; independent variable = nurses' academic degree

**TABLE S3** Tests of homogeneity of variances - years of experience.

|  | | Levene Statistic | df1 | df2 | Sig. |
| --- | --- | --- | --- | --- | --- |
| EBP Belief Mean Scores | Based on Mean | .568 | 1 | 180 | .452 |
|  | Based on Median | .268 | 1 | 180 | .605 |
|  | Based on Median and with adjusted df | .268 | 1 | 172.337 | .605 |
|  | Based on trimmed mean | .388 | 1 | 180 | .534 |
| EBP Organizational Culture Mean Scores | Based on Mean | .094 | 1 | 175 | .759 |
|  | Based on Median | .014 | 1 | 175 | .905 |
|  | Based on Median and with adjusted df | .014 | 1 | 172.969 | .905 |
|  | Based on trimmed mean | .074 | 1 | 175 | .785 |
| EBP Implementation Mean Scores | Based on Mean | .031 | 1 | 170 | .861 |
|  | Based on Median | .110 | 1 | 170 | .740 |
|  | Based on Median and with adjusted df | .110 | 1 | 165.652 | .740 |
|  | Based on trimmed mean | .099 | 1 | 170 | .753 |

*Note*. EBP = evidence-based practice; independent variable = categorized years of experience (1-40=1, 41-highest=2).

**TABLE S4** Normal distribution.

|  | Skewness | | Kurtosis | |
| --- | --- | --- | --- | --- |
|  | Statistic | Std. Error | Statistic | Std. Error |
| EBP Belief Mean Scores | -2.134 | .180 | 4.412 | .358 |
| EBP Organizational Culture Mean Scores | -.651 | .183 | -.149 | .363 |
| EBP Implementation Mean Scores | -1.528 | .185 | 3.611 | .368 |
| Years of Experience | 1.627 | .179 | 2.551 | .355 |

EBP = evidence-based practice.

**TABLE S5** ANOVA - academic degrees.

|  | | Sum of Squares | df | Mean Square | F | Sig. |
| --- | --- | --- | --- | --- | --- | --- |
| EBP Belief Mean Scores | Between Groups | 15028.175 | 3 | 5009.392 | 1.940 | .125 |
|  | Within Groups | 459723.825 | 178 | 2582.718 |  |  |
|  | Total | 474752.000 | 181 |  |  |  |
| EBP Organizational Culture Mean Scores | Between Groups | 7.009 | 3 | 2.336 | 2.136 | .097 |
|  | Within Groups | 189.218 | 173 | 1.094 |  |  |
|  | Total | 196.227 | 176 |  |  |  |
| EBP Implementation Mean Scores | Between Groups | 13158.378 | 3 | 4386.126 | 1.954 | .123 |
|  | Within Groups | 377105.122 | 168 | 2244.673 |  |  |
|  | Total | 390263.500 | 171 |  |  |  |

EBP = evidence-based practice.

**TABLE S6** ANOVA - years of experience.

|  | | Sum of Squares | df | Mean Square | F | Sig. |
| --- | --- | --- | --- | --- | --- | --- |
| EBP Belief Mean Scores | Between Groups | 1339.936 | 1 | 1339.936 | .509 | .476 |
|  | Within Groups | 473412.064 | 180 | 2630.067 |  |  |
|  | Total | 474752.000 | 181 |  |  |  |
| EBP Organizational Culture Mean Scores | Between Groups | .128 | 1 | .128 | .115 | .735 |
|  | Within Groups | 196.099 | 175 | 1.121 |  |  |
|  | Total | 196.227 | 176 |  |  |  |
| EBP Implementation Mean Scores | Between Groups | 3183.256 | 1 | 3183.256 | 1.398 | .239 |
|  | Within Groups | 387080.244 | 170 | 2276.943 |  |  |
|  | Total | 390263.500 | 171 |  |  |  |

EBP = evidence-based practice.

**TABLE S7** ANOVA - nursing roles.

|  | | Sum of Squares | df | Mean Square | F | Sig. |
| --- | --- | --- | --- | --- | --- | --- |
| EBP Belief Mean Scores | Between Groups | 8851.473 | 2 | 4425.736 | 1.700 | .186 |
|  | Within Groups | 465900.527 | 179 | 2602.796 |  |  |
|  | Total | 474752.000 | 181 |  |  |  |
| EBP Organizational Culture Mean Scores | Between Groups | 7.180 | 2 | 3.590 | 3.304 | .039 |
|  | Within Groups | 189.048 | 174 | 1.086 |  |  |
|  | Total | 196.227 | 176 |  |  |  |
| EBP Implementation Mean Scores | Between Groups | 11550.013 | 2 | 5775.007 | 2.577 | .079 |
|  | Within Groups | 378713.487 | 169 | 2240.908 |  |  |
|  | Total | 390263.500 | 171 |  |  |  |

EBP = evidence-based practice.

| **TABLE S8** Post hoc test EBP organizational culture multiple comparisons. | | | | | | | | |
| --- | --- | --- | --- | --- | --- | --- | --- | --- |
| Dependent Variable | | (I) Q5 | (J) Q5 | Mean Difference (I-J) | Std. Error | Sig. | 95% Confidence Interval | |
|  |  |  |  |  |  |  | Lower Bound | Upper Bound |
| **My organization has a supportive EBP Culture**  **My organization has resources to implement EBP culture**  **My organization provides EBP Mentors** | Games-Howell | 1* | 2 | .044 | .215 | .977 | -.47 | .56 |
|  |  |  | 3 | -.504 | .277 | .181 | -1.19 | .18 |
|  |  | 2* | 1 | -.044 | .215 | .977 | -.56 | .47 |
|  |  |  | 3 | -.548 | .314 | .201 | -1.31 | .22 |
|  |  | 3* | 1 | .504 | .277 | .181 | -.18 | 1.19 |
|  |  |  | 2 | .548 | .314 | .201 | -.22 | 1.31 |
|  | Games-Howell | 1 | 2 | -.116 | .209 | .845 | -.62 | .39 |
|  |  |  | 3 | -.704 | .287 | .053 | -1.42 | .01 |
|  |  | 2 | 1 | .116 | .209 | .845 | -.39 | .62 |
|  |  |  | 3 | -.588 | .322 | .174 | -1.37 | .20 |
|  |  | 3 | 1 | .704 | .287 | .053 | -.01 | 1.42 |
|  |  |  | 2 | .588 | .322 | .174 | -.20 | 1.37 |
|  | Games-Howell | 1 | 2 | -.189 | .208 | .638 | -.69 | .31 |
|  |  |  | 3 | -.688 | .293 | .065 | -1.41 | .04 |
|  |  | 2 | 1 | .189 | .208 | .638 | -.31 | .69 |
|  |  |  | 3 | -.499 | .326 | .289 | -1.29 | .30 |
|  |  | 3 | 1 | .688 | .293 | .065 | -.04 | 1.41 |
|  |  |  | 2 | .499 | .326 | .289 | -.30 | 1.29 |

*Note.* EBP = evidence-based practice.

*Legend:1 = Clinical Nurse; 2 = Advanced Practice Nurse; 3 = Nure Manager

**TABLE S9** Years of experience and EBP attributes.

|  | | EBP Belief Mean Scores | EBP Organizational Culture Mean Scores | EBP Implementation Mean Scores | Years of Experience |
| --- | --- | --- | --- | --- | --- |
| EBP Belief Mean Scores | Pearson Correlation | 1 | .317^**^ | .551^**^ | -.063 |
|  | Sig. (2-tailed) |  | <.001 | <.001 | .400 |
|  |  |  |  |  |  |
| EBP Organizational Culture Mean Scores | Pearson Correlation | .317^**^ | 1 | .497^**^ | .022 |
|  | Sig. (2-tailed) | <.001 |  | <.001 | .770 |
|  |  |  |  |  |  |
| EBP Implementation Mean Scores | Pearson Correlation | .551^**^ | .497^**^ | 1 | .013 |
|  | Sig. (2-tailed) | <.001 | <.001 |  | .867 |
|  |  |  |  |  |  |
| Years of Experience | Pearson Correlation | -.063 | .022 | .013 | 1 |
|  | Sig. (2-tailed) | .400 | .770 | .867 |  |
|  |  |  |  |  |  |
| Note. EBP = evidence-based practice.  ** Correlation is significant at the 0.01 level (2-tailed). | | | | | |
